# RSNA and BSTI grading systems of COVID-19 pneumonia: comparison of the diagnostic performance and interobserver agreement

**DOI:** 10.1186/s12880-021-00668-3

**Published:** 2021-10-04

**Authors:** Seyhmus Kavak, Recai Duymus

**Affiliations:** Department of Radiology, Gazi Yaşargil Training and Research Hospital, University of Health Sciences, Diyarbakır, Turkey

**Keywords:** Coronavirus, Computed tomography, RT-PCR, Guidelines of radiology

## Abstract

**Background:**

This study aimed to compare the performance and interobservers agreement of cases with findings on chest CT based on the British Society of Thoracic Imaging (BSTI) guideline statement of COVID-19 and the Radiological Society of North America (RSNA) expert consensus statement.

**Methods:**

In this study, 903 patients who had admitted to the emergency department with a pre-diagnosis of COVID-19 between 1 and 18 July 2020 and had chest CT. Two radiologists classified the chest CT findings according to the RSNA and BSTI consensus statements. The performance, sensitivity and specificity values of the two classification systems were calculated and the agreement between the observers was compared by using kappa analysis.

**Results:**

Considering RT-PCR test result as a gold standard, the sensitivity, specificity and positive predictive values were significantly higher for the two observers according to the BSTI guidance statement and the RSNA expert consensus statement (83.3%, 89.7%, 89.0%; % 81.2,% 89.7,% 88.7, respectively). There was a good agreement in the PCR positive group (κ: 0.707; p < 0.001 for BSTI and κ: 0.716; p < 0.001 for RSNA), a good agreement in the PCR negative group (κ: 0.645; p < 0.001 for BSTI and κ: 0.743; p < 0.001 for RSNA) according to the BSTI and RSNA classification between the two radiologists.

**Conclusion:**

As a result, RSNA and BSTI statement provided reasonable performance and interobservers agreement in reporting CT findings of COVID-19. However, the number of patients defined as false negative and indeterminate in both classification systems is at a level that cannot be neglected.

**Supplementary Information:**

The online version contains supplementary material available at 10.1186/s12880-021-00668-3.

## Background

The diagnosis of COVID-19 is primarily carried out by the reverse transcription polymerase chain reaction (RT-PCR), which is a nucleic acid amplification test (NAAT) of SARS-CoV-2 RNA sampled from the upper respiratory tract [1]. NAATs detect SARS-CoV-2 RNA in patient samples, and they are highly specific. Although they are able to detect even low levels of viral RNAs, the sensitivity of these tests in clinical setting is likely to depend on the type and quality of the sample obtained, the duration of the disease at the time point of the test and the individual test. Their estimated false-negative rates ranges from 5 to 40% [2, 3]. In early or mild disease, chest radiographies may be normal. Chest computed tomography (CT) is more sensitive than chest radiography, and some CT findings can be considered as characteristical to COVID-19. However, the absence of typical signs in CT or presence of atypical manifestations of this disease can not completely eliminate or rule out diagnosis of COVID-19 [4, 5].

There are many articles and case reports trying to identify chest CT findings in COVID-19 disease. Different findings and reports presented every day for discussion in the light of the existing literature. Although, the common opinion in the radiology society is to reject using chest CT as a diagnostic tool in COVID-19, a growing number of studies classifying CT findings of the disease exists. Currently, there are many studies focusing on chest CT findings, changes of these findings throughout the disease period, and on the practices of radiologists using these findings in differential diagnosis of COVID-19 [6–10].

COVID-19 may present with a broad spectrum of findings ranging from ground-glass opacities (GGO) to an apparent pneumonia [11]. Furthermore, additional findings including linear or curvilinear bands, nodular densities, GGO and consolidations, and cobblestone pattern that can be seen in other viral or bacterial pneumonia, drug toxicities, inhalation exposure and some systemic diseases [12–14]. Due to similar imaging patterns of different etiologies other than COVID-19, diagnostic process may be complicated, relatively. It can also lead to stress and loss of labor for patients and their relatives, as well as healthcare professionals [15]. For a better diagnostic process, standardization of reporting and direct contact with the attending physician are important parameters to increase productivity. Many institutes and radiology societies proposed classifications that may reveal presence, severity and may predict prognosis of the disease based on CT findings but it has yet to be attained to a clear consensus. In order to help radiologists recognize and correctly identify chest CT findings in COVID-19 disease, similar guidelines that are slightly differing from each other have been confirmed and published by associations such as Society of Thoracic Radiology, Radiology Society of North America (RSNA), British Society of Thoracic Imaging (BSTI), and Dutch Radiological Society (Additional file 1: Tables E1–2) [16–18]. 

In this study, it was aimed to reveal imaging findings of patients admitted to hospital with a pre-diagnosis of COVID-19 and of those who have suspicious findings in chest CT in company with their epidemiological data, to classify the patients in terms of findings of COVID-19 based on the BSTI guidance statement and the RSNA expert consensus statement, and to compare both of classification systems.

## Methods

This monocentric, retrospective study has been approved by the COVID-19 Scientific Research Committee of the Republic of Turkey Ministry of Health and Sağlık Bilimleri Üniversitesi Gazi Yaşargil Training and Research Hospital Clinical Trials Ethics Board in accordance with the Declaration of Helsinki. The approval of the study was granted by the Institutional Ethics Committee (Gazi Yaşargil Training and Research Hospital, decision no: 588/2020). The informed consent form was waived because of no risk owing to the study and there were no adversely affected subjects or groups.

### Patient selection

**Fig. 1:**
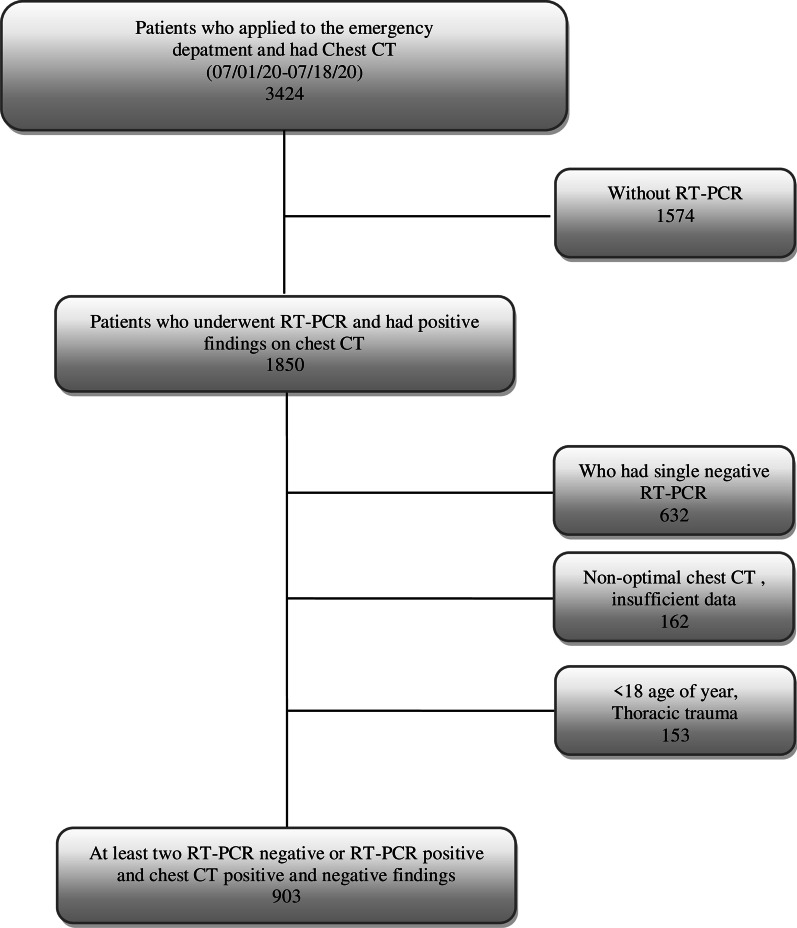
Flow chart illustrating the patient selection

In this study, 903 of 5708 patients who applied to Gazi Yaşargil Training and Research Hospital, Sağlık Bilimleri Üniversitesi (Diyarbakır, Turkey) with a history of contact or symptoms of COVID-19 and underwent chest CT examination between 1 and 18 July 2020 were included (see flow chart) (Fig. 1). RT-PCR test was performed on a total of 3561 patients and the result was recorded as positive in 637 patients. Of the patients who were positive, 612 who met the appropriate criteria and 291 patients with a negative test were included in the study.

Among the most common reasons for admission to the emergency room were symptoms of high fever, cough, weakness, shortness of breath, and contact history. Subjects had been diagnosed according to the COVID-19 (SARS-CoV-2 Infection) Guide (Study of Scientific Board, Ministry of Health, Republic of Turkey, April 14, 2020). After obtaining their anamnesis and physical examination, laboratory tests including RT-PCR, and radiological imagings of respiratory system were conducted. Those who had positive RT-PCR test and pathological findings in thorax CT were diagnosed as COVID-19. On the other hand, subjects with pathological findings in thorax CT, but with two consecutive RT-PCR test negativity obtained at least 24 h later were accepted as non COVID-19.

### Obtainment of thorax CT imagings and technical parameters

All the examinations were conducted using 16- and 64-slice multi-slice CT scanners [Emotion 16, Somatom Sensation 64 (Siemens Healthineers, Erlangen, Germany)]. All patients were admitted to the imaging unit with a surgical mask on. Radiology technicians got the imagings with an N95 mask, face-shield, gloves, and disposable apron. After each implementation, the surfaces had been disinfected by sodium hypochlorite solution and the room was ventilated for at least 10 min. CT examinations were non-contrast, and were performed in supine position and in deep-inspiration breath-hold. CT imaging protocol was programmed in 120 kV, 80 mA, 1 mm slice thickness, and with a standard or lower dose adjusted by using automatic dosing. Axial images were reconstructed with a slice thickness of 1.5 mm to obtain coronal images. Window settings were selected as a window level of − 600 HU and a window width of 1500 HU for lung parenchyma and a window level of 40 HU and a window width of 350 HU for mediastinum.

### Interpretation of imagings

Thoracic CT examinations were blindly reported in terms of RT-PCR test results by two radiologists with a 14 and 15 years of experience, respectively (S.K. and R.D.).

**Fig. 2:**
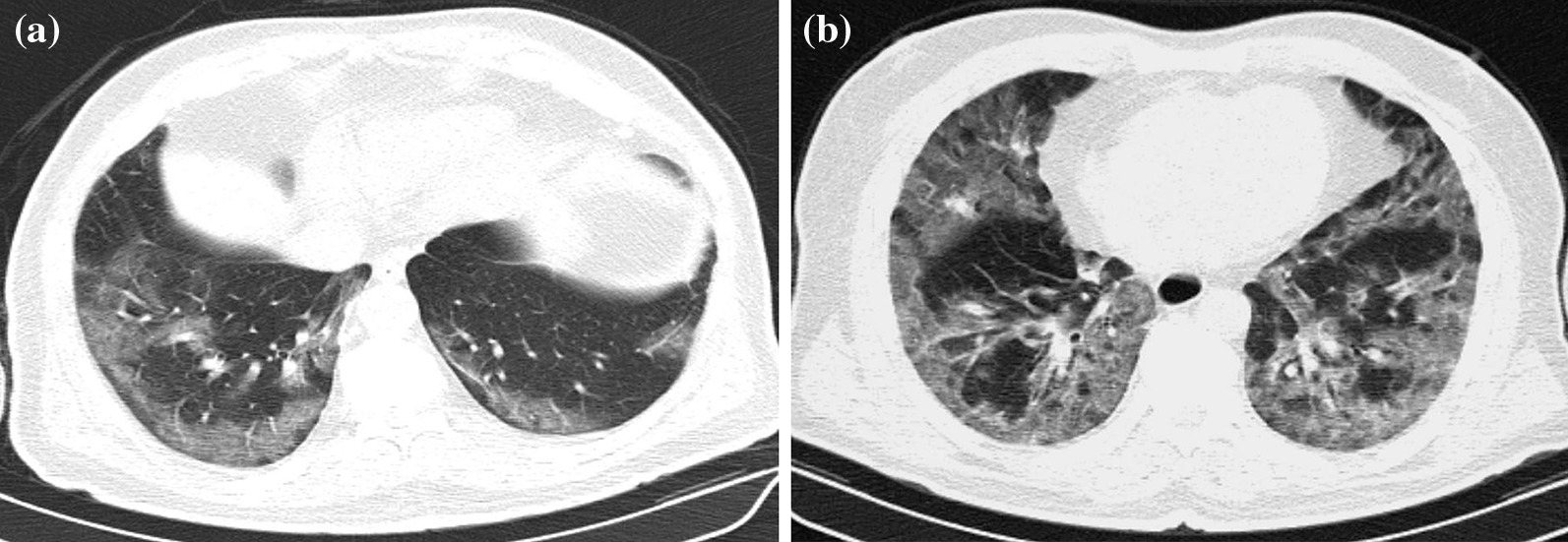
**a, b** Case examples defined in the classic COVID-19 for the BSTI guidance statement, and in the typical category for the RSNA expert consensus statement. **a** An axial chest CT image of a 68-year-old male with COVID-19 shows peripheral, bilateral, multifocal GGOs. **b** An axial chest CT image of a 64-year-old male with COVID-19 shows peripheral, bilateral, multifocal GGOs

**Fig. 3:**
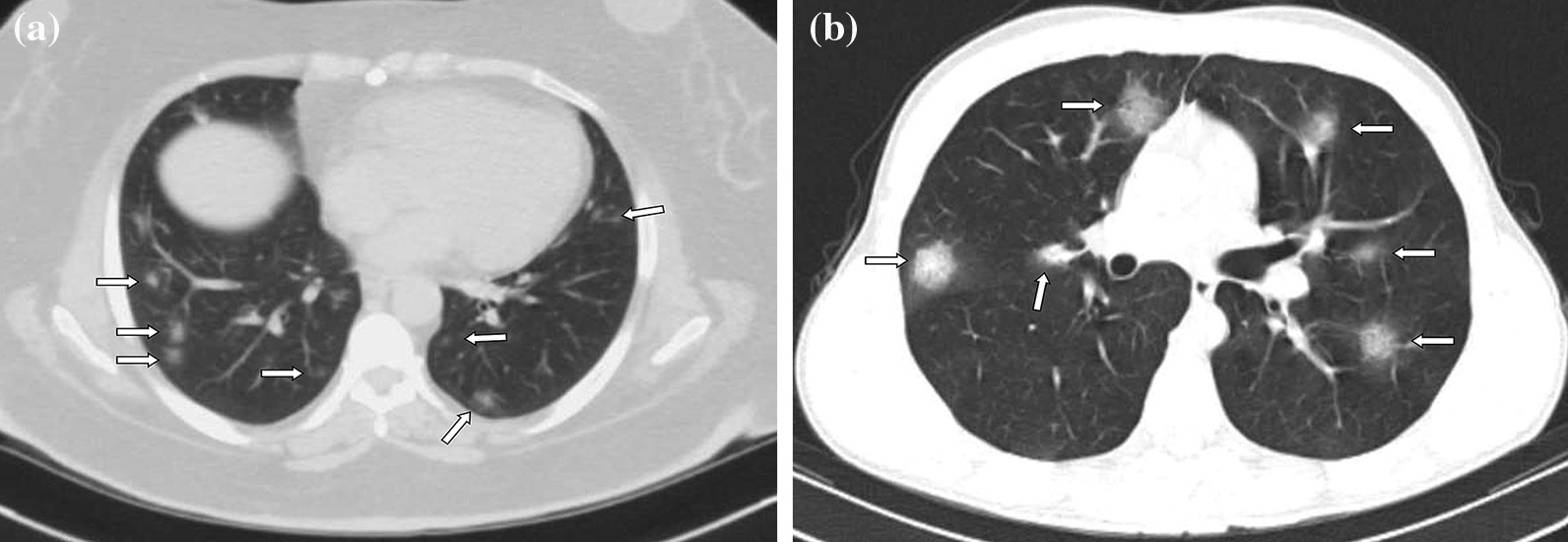
**a, b** Case examples defined in the probable COVID-19 for the BSTI guidance statement, and in the typical category for the RSNA expert consensus statement. **a** An axial chest CT image of a 46-year-old female with COVID-19 shows peripheral, bilateral, multifocal, rounded GGOs (arrows). **b** An axial chest CT image of a 19-year-old male with COVID-19 shows peripheral, bilateral, multifocal, rounded GGOs (arrows)

**Fig. 4:**
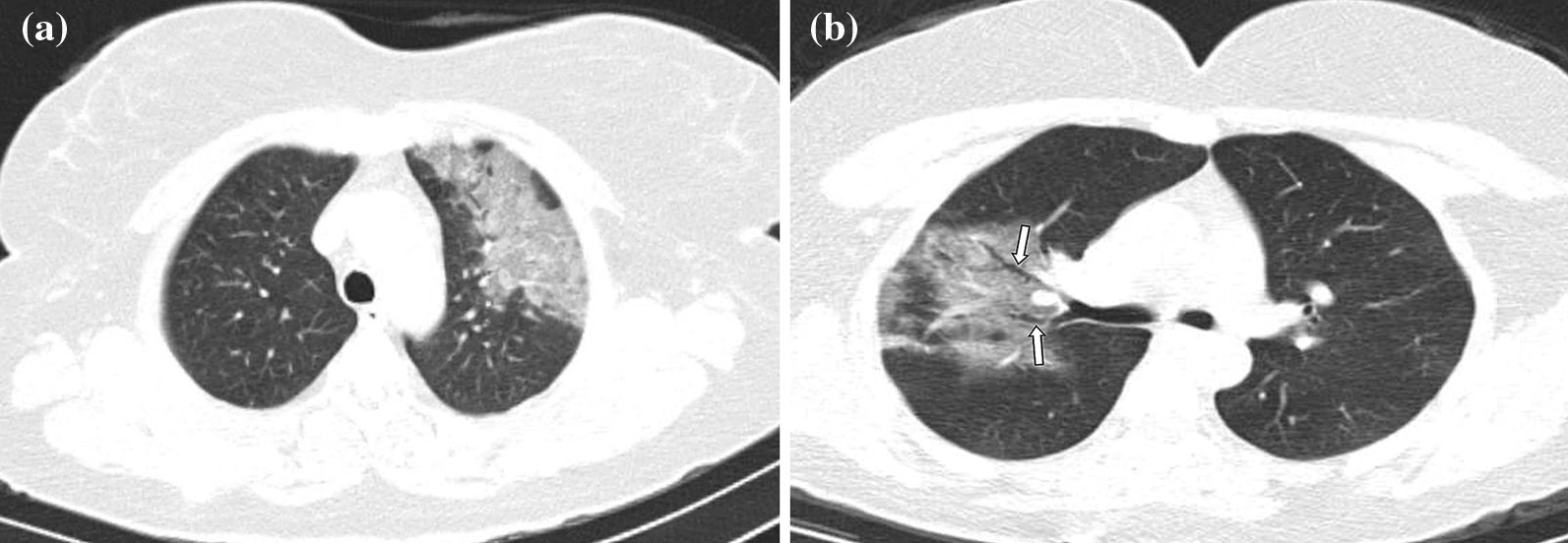
**a, b** Case examples defined in the classic COVID-19 for the BSTI guidance statement, and in the indeterminate category for the RSNA expert consensus statement. **a** An axial chest CT image of a 65-year-old female with COVID-19 shows segmental (the left upper lobe) GGOs and bronchial wall thickening. **b** An axial chest CT image of a 41-year-old female with COVID-19 shows segmental GGOs and vascular enlargement with bronchial wall thickening (arrows)

**Fig. 5:**
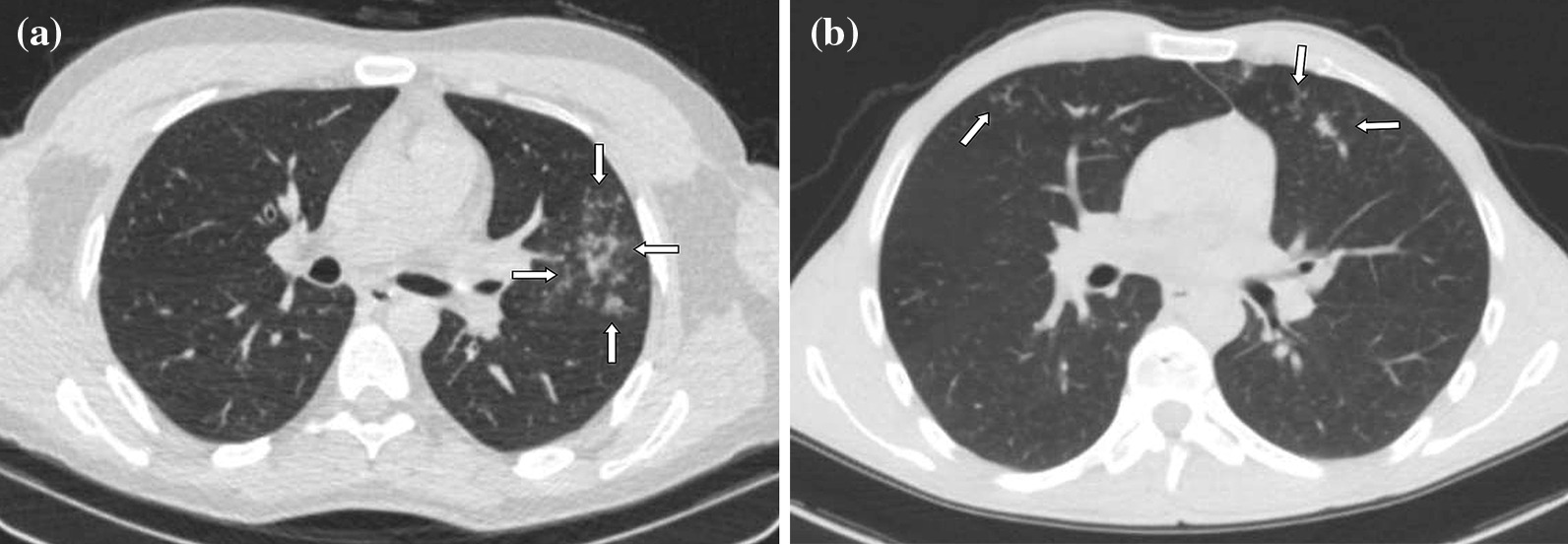
**a, b** Case examples defined in the non-COVID for the BSTI guidance statement, and in the atypical category for the RSNA expert consensus statement. **a** An axial chest CT image of a 36-year-old male without COVID-19 shows centrilobular nodules without GGOs in the left upper lobe (arrows). The final diagnosis was bronchial pneumonia. **b** An axial chest CT image of a 37-year-old male without COVID-19 shows centrilobular nodules and tree-in-bud appearance without GGOs in the right middle and left lingula segment (arrows). The final diagnosis was of lung tuberculosis

**Fig. 6:**
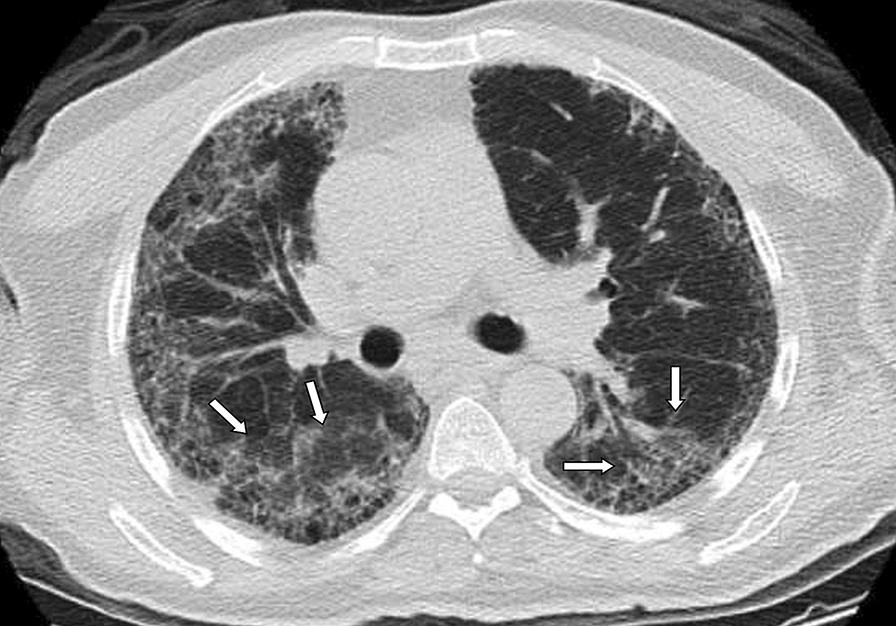
Case example defined in the indeterminate for the BSTI guidance statement, and in the typical category for the RSNA expert consensus statement. An axial chest CT image of a 68-year-old male without COVID-19 shows peripheral multifocal GGOs (arrows) in the background of moderate emphysema. The final diagnosis was usual interstitial pneumonia

Although, recurring chest CT imagings were performed in some patients because of various reasons such as sudden deterioration in clinical course or laboratory values, first admission chest CTs were considered for evaluation. Chest CTs were assessed in terms of presence of GGOs, consolidations, nodular densities, crazy-paving pattern, reticulation, subpleural bands, interlobular septal thickening, halo and reversed halo signs, enlarged vascular sign at the level of lesion, bronchial wall changes, traction bronchiectasis, enlarged mediastinal lymph node, etc., which are considered to be related to COVID-19. In addition, other findings atypical to COVID-19 such as infectious cavitation, tree-in-bud pattern, lobar pneumonia and consolidation, and pleural effusion were also evaluated. Interpretation was based on the classification recommended by RSNA expert consensus statement and BSTI guidance statement, which was prepared based on chest CT findings of COVID-19 [16, 17]. The findings were recorded as typical, indeterminate, atypical and negative according to the RSNA guidelines, and as classical COVID-19, probable COVID-19, indeterminate and non-COVID-19 according to the BSTI classification (Figs. 2, 3, 4, 5, 6). The correlation between the two radiologists and the data obtained according to each classification system were compared.

### Statistical analysis

The Statistical Package for Social Sciences (SPSS for Windows 24.0, SPSS Inc, Chicago, Illinois, USA) computer program was used for statistical analyses. Number, percentage, mean ± standard deviation (SD) values were used for descriptive statistics. Shapiro–Wilk test was used for assumptions of normality and homogeneity of variances. Chi-square test was used for comparison of categorical data. The results were evaluated using hazard ratio and with 95% confidence interval. Kappa statistics were used to determine the interobservers reliability. The degree of interobservers agreement was considered with the following interval of kappa: 0–0.20 for poor, 0.21–0.40 for fair, 0.41–0.60 for moderate, 0.61–0.80 for good, and 0.81–1.00 for excellent agreement. In order to measure the diagnostic power of both classification systems, the receiver operating curve (ROC) analysis was also performed. Sensitivity, specificity, positive predictive value (PPV), and negative predictive value (NPV) were calculated by setting different cut-off points for each criterion. In these analyses, p values < 0.05 were considered statistically significant.

## Results

### Demographic findings

In this retrospective study, 903 inpatients and outpatients were included. A total of 459 (50.8%) of the patients were female, 444 (49.2%) were male, and the age range was between 18 and 95 years (mean age 48.66 ± 16.87). A Rh (+) was detected in 368 patients (40.8%), while the second most common group was 0 Rh (+) in 217 patients (24.1%). A total of 366 (40.5%) patients had comorbid disease. Hypertension (28.5%) was the most common comorbidity, followed by heart failure and coronary artery disease, chronic obstructive pulmonary disease and diabetes mellitus (Table 1). The mean hospitalization period was 9 days (1–47); 860 (95.2%) patients were discharged, and 43 (4.8%) died.

**Table 1:** Distribution of demographic characteristics, comorbid diseases and blood groups

Variable	N	%
*Gender*		
Female	459	50.8
Male	444	49.2
*Blood group*		
A Rh(+)	368	40.8
B Rh(+)	155	17.2
0 Rh(+)	217	24.1
*Survival*		
Discharge	860	95.2
Died	43	4.8
*RT-PCR*		
Positive	612	67.8
Negative	291	32.2
*Comorbid disease*	366	40.5
Hypertension	257	28.5
DM^a^	90	10.0
CAD^b^/CHF^c^	136	15.1
COPD^d^	112	12.4
Immuno suppression	8	0.9
Malignancy	6	0.7
CKD^e^	20	2.2

^a^Diabetes mellitus

^b^Coronary artery disease

^c^Congestive heart failure

^d^Chronic obstructive pulmonary disease

^e^Chronic kidney disease

### Radiological findings

In accordance with the literature, the most common finding was GGOs, which were detected in 685 patients (75.8%). Ground-glass opacities were mostly patchy patterns and were peripherally localized [8, 10, 19]. In addition, they had a multilobar distribution, and they were mostly located at the level of the lower lobes and in the posterior zone. Ground-glass opacities or consolidation were detected in 45 patients (5.0%) at the isolated upper and middle zone levels. There was consolidation together with GGO in 276 (30.6%) subjects. Ground-glass opacities were bilaterally observed in the lungs in 561/685 subjects (81.9%). The number of subjects with unilateral right lung involvement was 75 (10.9%), while the number of those with unilateral left lung involvement was 49/685 (7.2%). The most common concomitant finding was enlarged vascular sign (≥ 3 mm) at the lesion level in 414/685 patients (60.4%), followed by interlobular septal thickening, crazy-paving pattern, pleural and reticular bands, traction bronchiectasis, atelectasis and air bronchogram. The tree-in-bud pattern was detected in 39 patients (4.3%), centrilobular nodules in 50 patients (5.5%) and lobar consolidation in 33 patients (3.7%).

There was good agreement in the PCR-positive and PCR-negative groups according to the BSTI classification between the two radiologists (κ: 0.707; p < 0.001, κ: 0.645; p < 0.001, respectively; see Table 2). Accordingly, good agreement in the PCR-positive and PCR-negative groups (0.716; p < 0.001, κ: 0.743; p < 0.001, respectively; see Table 3) was found according to the RSNA classification.

**Table 2:** Evaluation of agreement between two radiologists in RT-PCR positive and negative patients according to BSTI guidance statement

RT-PCR		BSTI guidance statement		Observer B				Test value Kappa (κ); *p*
				1	2	3	4	
Positive	Observer A	1-Classical COVID-19	N %	357 91.5	32 8.2	1 0.3	0	0.707; < 0.001
		2-Probable COVID-19	N %	36 30.0	73 60.8	8 6.7	3 2.5	
		3-Indeterminate	N %	0	7 11.7	43 71.7	10 16.7	
		4-COVID-19 exclusion	N %	0	0	4 9.5	38 90.5	
Negative	Observer A	1-Classical COVID-19	N %	13 100.0	0	0	0	0.645; < 0.001
		2-Probable COVID-19	N %	0	14 82.4	2 11.8	1 5.9	
		3-Indeterminate	N %	0	1 2.2	24 53.3	20 44.4	
		4-COVID-19 exclusion	N %	0	1 0.5	23 10.6	192 88.9

**Table 3:** Evaluation of agreement between two radiologists in RT-PCR positive and negative patients according to RSNA expert consensus statement

RT-PCR		RSNA expert consensus statement		Observer B				Test value Kappa (κ); *p*
				1	2	3	4	
Positive	Observer A	1-Typical COVID-19	N %	483 97.2	11 2.2	3 0.6	0	0.716; < 0.001
		2-Indeterminate	N %	12 19.0	34 54.0	13 20.6	4 6.3	
		3-Atypical COVID-19	N %	1 5.9	8 47.1	1 5.9	7 41.2	
		4-Negative for Pneumonia	N %	0	1 2.9	1 2.9	33 94.3	
Negative	Observer A	1-Typical COVID-19	N %	27 90.0	2 6.7	1 3.3	0	0.743; < 0.001
		2-Indeterminate	N %	0	22 57.9	16 42.1	0	
		3-Atypical COVID-19	N %	1 1.0	22 21.0	76 72.4	6 5.7	
		4-Negative for pneumonia	N %	0	0	5 4.2	113 95.8

**Table 4:** Comparison of patients' classification of two observers in terms of COVID-19 according to BSTI guidance statement and RSNA expert consensus statement

RT-PCR observer A				RSNA expert consensus statement			Test Value X^2^; *p*
				Typical	Indeterminate	Atypical or Negative	
Positive	BSTI guidance statement	Classical or probable COVID-19	N %	497 97.4	10 2.0	3 0.6	872.18; < 0.001
		Indeterminate	N %	0	52 86.7	8 13.3	
		COVID-19 exclusion	N %	0	1 10.0	9 90.0	
Negative	BSTI guidance statement	Classical or probable COVID-19	N %	30 100.0	0	0	785.64; < 0.001
		Indeterminate	N %	0	29 64.4	16 35.6	
		COVID-19 exclusion	N %	0	9 4.2	207 95.8	

RT-PCR observer B				RSNA expert consensus statement			Test value X^2^; *p*
				Typical	Indeterminate	Atypical or negative	
Positive	BSTI guidance statement	Classical or probable COVID-19	N %	495 98.0	9 1.8	1 0.2	881.49; < 0.001
		Indeterminate	N %	1 1.8	44 78.6	11 19.6	
		COVID-19 exclusion	N %	0	1 5.3	18 94.7	
Negative	BSTI guidance statement	Classical or probable COVID-19	N %	28 96.5	1 3.5	0	893.98; < 0.001
		Indeterminate	N %	0	41 83.7	8 16.3	
		COVID-19 exclusion	N %	0	4 1.9	209 98.1	

X^2^: Pearson Chi-Square

According to the assessment of thorax CT scans of RT–PCR-positive subjects by Observer A based on the RSNA guidelines, 497 (81.2%) of the subjects were typical, 63 (10.3%) were indeterminate, 17 (2.8%) were atypical and 35 (5.7%) were negative. However, according to the BSTI guidelines, 390 (63.7%) were classic COVID-19, 120 (19.6%) were probable COVID-19, 60 (9.8%) were indeterminate, and 42 (6.9%) were non-COVID-related (Table 4).

When using the RT–PCR test result as the gold standard, Observer A yielded a sensitivity of 83.3%, a specificity of 89.7%, a PPV of 89.0%, an NPV of 84.3%, an accuracy 86.5%, and Likelihood Ratio (LR+) values of 8.1 for the Classic and Probable COVID-19 category of the BSTI guide statement with a p value of < 0.001. According to the same observer, for the typical COVID-19 category, the sensitivity of the RSNA expert consensus statement was 81.2%, the specificity was 89.7%, the PPV was 88.7%, the NPV was 82.7%, the accuracy was 85.4%, the LR + value was 7.9, and the p value was < 0.001 (Table 5).

**Table 5:** Sensitivity, specificity, and positive and negative predictive values, accuracy ratio and likelihood ratios according to BSTI and RSNA criteria

	Sensitivity	Specificity	PPV*	NPV*	Accuracy*	LR^+^	*p* value
*RSNA-typical COVID-19*							
Observer A	81.2%	89.7%	88.7%	82.7%	85.4%	**7.9**	** < 0.001**
Observer B	81.0%	90.4%	89.2%	82.6%	85.6%	**8.4**	** < 0.001**
BSTI-classic COVID-19							
Observer A	63.7%	95.5%	93.5%	72.5%	79.6%	**14.1**	** < 0.001**
Observer B	64.2%	95.5%	93.6%	72.8%	79.9%	**14.3**	** < 0.001**
BSTI-classic and probable COVID-19							
Observer A	83.3%	89.7%	89.0%	84.3%	86.5%	**8.1**	** < 0.001**
Observer B	82.5%	90.0%	89.2%	83.7%	86.3%	**8.2**	** < 0.001**

*PPV* positive predictive value, *NPV *negative predictive value

*Reverse transcription polymerase chain reaction positive and negative patient numbers were equalized while calculating PPV, NPV and accuracy ratio

^+^Likelihood ratios

**Fig. 7:**
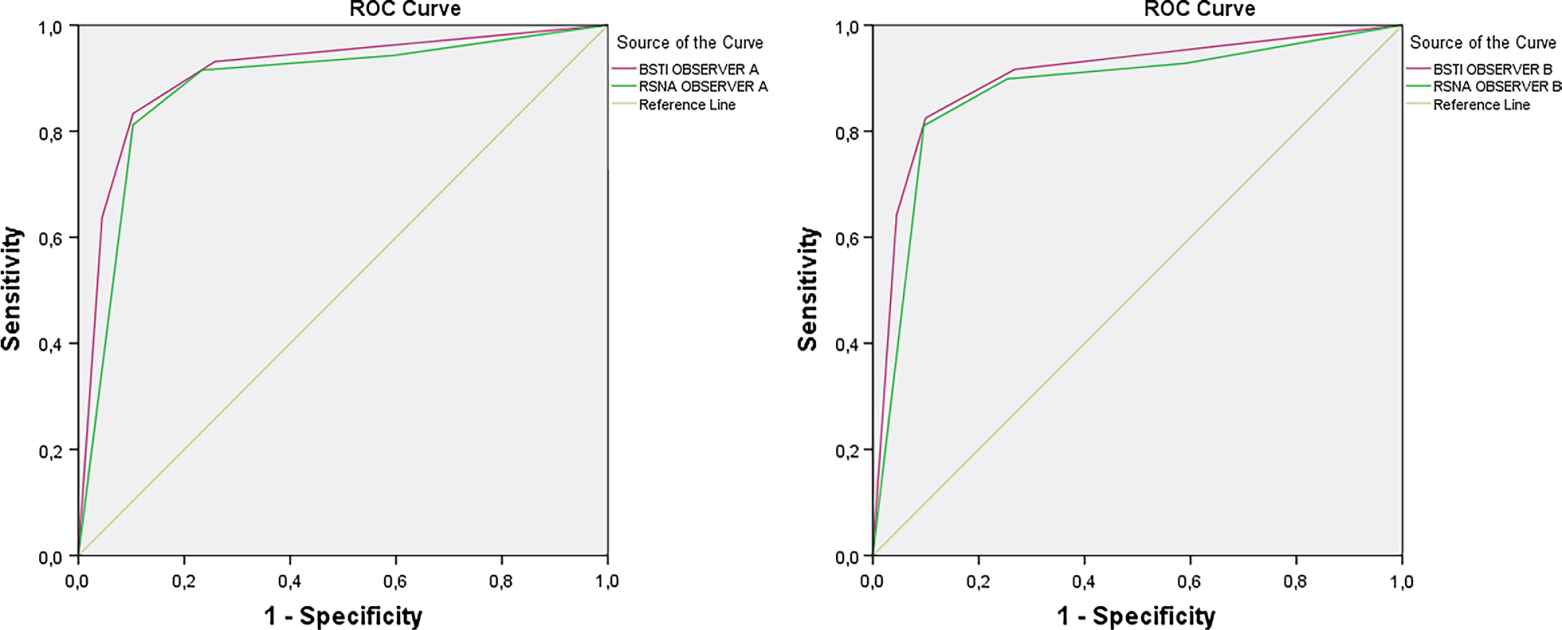
**a**, **b** ROC curves of both observers to predict COVID-19 according to BSTI and RSNA classification. **a** For observer A and **b** for observer B

In addition, the diagnostic performances of both observers in RT–PCR-positive and RT–PCR-negative groups according to the BSTI guidance statement and RSNA expert consensus statement were determined by ROC analysis, and the area under the curve (AUC) was calculated. Although the diagnostic performance of both classification systems was similar, the power of the BSTI guidance statement was higher than that of the RSNA expert consensus statement for both observers (observer A for BSTI: AUC 0.910 (95% CI 0.889–0.932) and for RSNA: AUC 0.884 (95% CI 0.858–0.909); observer B for BSTI: AUC 0.903 (95% CI 0.881–0.925) and for RSNA: AUC 0.876 (95% CI 0.850–0.902)) (Fig. 7).

## Discussion

As a common finding and one of the most important descriptive findings in COVID-19 pneumonia, peripheral and bilateral GGOs are the most common finding in this study and have been considered the most important parameter for the identification of the disease in the classification systems of RSNA and BSTI. In accordance with the literature, the predominance of the posterior zone of GGOs was observed in this study. Other important findings that both classification systems use in predicting the disease include consolidation co-occurring with GGOs, findings of organized pneumonia and halo signs [8, 11, 16, 20, 21]. In terms of these findings, the success of the diagnostic process in both classification systems was similar. The findings classified as typical in the RSNA guidelines and the findings classified as classic COVID-19 and probable COVID-19 in the BSTI guidelines match to a great extent. The presence of positive radiological findings that can indicate the disease and thus may contribute to the detection of the disease by repeating the test in case of RT–PCR test false negativity can lead to an earlier treatment and therefore reduced morbidity and mortality. In addition, in a recently published study, it was reported that structured radiological (SR) reporting for chest CT is more reliable than conventional radiological (CR) reporting in COVID-19, as in many diseases [22].

The number of patients identified by both radiologists in RT–PCR positive patients in the typical COVID-19 category of RSNA and the classic and probable COVID-19 categories by BSTI were similar (Radiologist A: 497/612, 81.2% for RSNA and 510/612, 83.3% for BSTI, Radiologist B: 496/612, 81.2% for RSNA and 505/612, 82.5% for BSTI, respectively). In our study, the correlation between two radiologists in RT–PCR-positive patients was k: 0.716 for RSNA and k: 0.707 for BSTI. De Jaegere et al., in their study of 96 patients, compared the RSNA and CO-RADS classifications and found that they had similar identification power. In the assessment between the three readers, 62.2% (28/45), 37.8% (17/45), and 44.4% (20/45) in the typical category for RSNA were identified, respectively. Considering the correlation between the readers for RSNA, kappa values ranged from 0.564 to 0.663 and were similar to our study [23]. In two other studies, according to the RSNA expert consensus statement, it has been reported that interobserver agreement varies between good and excellent (K = 0.822–0.924). In both studies, interobserver agreement was found to be significantly high in typical and negative categories for RSNA, whereas it was found to be poor or moderate in indeterminate and atypical categories [24, 25]. In these studies, the high number of patients in the negative category for RSNA may have led to a partial increase in interobserver agreement. Conversely, in our study, due to the low number of patients in the negative category for RSNA, the interobserver agreement may have been found to be lower.

Regarding the diagnostic power of both classification systems, the sensitivity, specificity and PPV values were high, and the diagnostic power of BSTI was found to be slightly higher than that of RSNA (83.3%, 89.7%, 89.0% for BSTI, 81.2%, 89.7%, 88.7% for RSNA). In a similar study, Inui et al. compared the typical category of RSNA with the classic COVID-19 and possible COVID-19 categories of BSTIs in terms of sensitivity, specificity, PPV, and NPV. They reported that the sensitivity was higher in RSNA (73.5% versus 71.3%), whereas the specificity was higher in BSTI (87.3% versus 82.8%)[26]. Ciccarese et al. reported that analysis of the pattern distribution was 'typical' (n = 151/211), a sensitivity of 71.6%, a specificity of 91.6%, and a PPV of 87.8% for COVID-19. There was excellent agreement between the two observers for typical and negative results (84.7% and 91.3%, respectively) [27]. Özer et al. reported high sensitivity and specificity values in the typical category for RSNA. In addition, ROC analysis was performed in RT–PCR-positive and RT–PCR-negative patients in this study, and the AUC was reported to be 0.878 (95% CI 0.852–0.903)[23].

However, very small GGOs that are peripheral and not round and GGOs with unilateral involvement that are defined in the indeterminate group in the RSNA classification can be classified as probable COVID-19 in the classification of BSTI. Another difference between the two classification systems is that although some diseases, such as pre-existing interstitial pneumoniae, are not considered by the RSNA expert consensus statement, the BSTI guidance statement suggested an increase of one degree in those patients [16, 17]. In both classification systems, the findings that were considered typical or highly suspected for COVID-19 disease were not detected in a significant number of patients, and they were classified in the indeterminate category. In RT–PCR-positive patients, 10 (15.9%) of 63 patients identified by observer A in the indeterminate group according to the RSNA classification system were in the classical or probable COVID-19 group in the BSTI classification. The cases considered indeterminate according to both RSNA and BSTI classifications in RT–PCR-positive subjects were examined in detail, and it was seen that the majority [40/63 (63.5%) and 39/60 (65.0%), respectively] were in the early stage of the disease. In light of the available data, due to the nature of the disease, there may be no CT findings or ambiguous CT findings at the early stage of the disease, although ground-glass opacities might present along with consolidation and other findings in later stages [9]. CT findings in COVID-19 disease have a wide spectrum depending on the epidemiological characteristics of the patient (age, sex, race), presence of comorbid disease, any concomitant parenchymal lung disease, duration and stage of the disease, and severity of the disease [6, 28]. Higher percentages of patients classified as indeterminate in both classification systems can be explained by that.

In RT–PCR-negative subjects, chest CT findings in a considerable number of subjects were congruent with COVID-19 according to both classification systems (typical category 30/291 (10.3%) for RSNA and 30/291 (10.3%) for classical and probable categories for BSTI). In particular, a high number of false-positive subjects can cause problems in the diagnostic process. When an indeterminate group was added to the false-positive group, the rate of exclusion of COVID-19 among RT–PCR-negative subjects was calculated to be 76.6% for RSNA and 74.3% for BSTI, and in that respect, both classifications were approximate. One reason for the relatively low rates may be the lower number of cases without chest CT findings in the positive group and in the negative group. In addition, in our study, the correlation between two radiologists in RT–PCR-negative patients was k: 0.743 for RSNA and k: 0.645 for BSTI. Based on both classification systems, a good correlation was found between the operators in RT–PCR-positive and RT–PCR-negative subjects.

The most important limitation of this study is that few subjects with aetiologies other than COVID-19 that may show similar CT findings were included. Therefore, studies with larger cohorts including other aetiologies might reveal more accurate specificity and sensitivity rates.

## Conclusions

Many radiology associations disapprove of the use of chest CT examination in the diagnostic process of COVID-19. However, currently, it is widely used for diagnostic purposes and for predicting and monitoring the course of the disease. Since it is a newly defined disease, complete detection and accurate identification of CT findings, eliminating other aetiologies that lead to similar findings, and standardization in reporting to help radiologists and other clinicians are of importance. Several radiology societies have therefore proposed a classification system for COVID-19 based on CT findings to create a reliable and accurate diagnostic process for radiologists. In this study, chest CT findings of RT–PCR-positive and RT–PCR-negative subjects were revealed in detail. In conclusion, based on these findings, the guidelines proposed by RSNA and BSTI were successful in detecting COVID-19, and they can be reliable references for radiologists since both present similar results.

## Supplementary Information

**Additional file 1**. Structured thorax CT report recommended by the BSTI in COVID-19 pneumonia.

**Additional file 2**. Expert consensus statement on reporting of RSNA-recommended chest CT findings related to COVID-19.

## Data Availability

Not applicable.

## Abbreviations

BSTI: British Society of Thoracic Imaging
CO-RADS: COVID-19 Reporting and Data System
COVID-19: Coronavirus disease 2019
CT: Computed tomography
GGO: Ground-glass opacities
NAAT: Nucleic acid amplification test
RSNA: Radiology Society of North America
RT-PCR: Reverse transcription polymerase chain reaction
SD: Standard deviation
WHO: World Health Organization

## References

[1] Fang FC, Naccache SN, Greninger AL (2020). The laboratory diagnosis of COVID-19-frequently-asked questions. Clin Infect Dis.

[2] Wang W, Xu Y, Gao R, Lu R, Han K, Wu G (2020). Detection of SARS-CoV-2 in different types of clinical specimens. JAMA.

[3] Yu F, Yan L, Wang N, Yang S, Wang L, Tang Y (2020). Quantitative detection and viral load analysis of SARS-CoV-2 in infected patients. Clin Infect Dis.

[4] Wong HYF, Lam HYS, Fong AH, Leung ST, Chin TW, Lo CSY (2020). Frequency and distribution of chest radiographic findings in patients positive for COVID-19. Radiology.

[5] ACR Recommendations for the use of Chest Radiography and Computed Tomography (CT) for Suspected COVID-19 Infection. https://www.acr.org/Advocacy-and-Economics/ACR-Position-Statements/Recommendations-for-Chest-Radiography-and-CT-for-Suspected-COVID19-Infection. Accessed 1 April 2020.

[6] Pan F, Ye T, Sun P, Gui S, Liang B, Li L (2020). Time course of lung changes on chest CT during recovery from 2019 Novel Coronavirus (COVID-19) Pneumonia. Radiology.

[7] Kong W, Agarwal P (2020). Chest imaging appearance of COVID-19 infection. Radiol Cardiothorac Imaging.

[8] Bai HX, Hsieh B, Xiong Z, Halsey K, Choi JW, Tran TML (2020). Performance of radiologists in differentiating COVID-19 from viral pneumonia on chest CT. Radiology.

[9] Bernheim A, Mei X, Huang M, Yang Y, Fayad ZA, Zhang N (2020). Chest CT findings in Coronavirus Disease-19 (COVID-19): relationship to duration of infection. Radiology.

[10] Chung M, Bernheim A, Mei X, Zhang N, Huang M, Zeng X (2020). CT imaging features of 2019 Novel Coronavirus (2019-nCoV). Radiology.

[11] Salehi S, Abedi A, Balakrishnan S, Gholamrezanezhad A (2020). Coronavirus disease 2019 (COVID-19): a systematic review of imaging findings in 919 patients. AJR Am J Roentgenol.

[12] Franquet T (2011). Imaging of pulmonary viral pneumonia. Radiology.

[13] Cleverley JR, Screaton NJ, Hiorns MP, Flint JD, Muller NL (2002). Drug-induced lung disease: high-resolution CT and histological findings. Clin Radiol.

[14] Ellis SJ, Cleverley JR, Müller NL (2000). Drug-induced lung disease: high-resolution CT findings. AJR Am J Roentgenol.

[15] Obadina ET, Torrealba JM, Kanne JP (2013). Acute pulmonary injury: high-resolution CT and histopathological spectrum. Br J Radiol.

[16] Simpson S, Kay FU, Abbara S, Bhalla S, Chung JH, Chung M (2020). Radiological Society of North America Expert Consensus Statement on Reporting Chest CT Findings Related to COVID-19. Endorsed by the Society of Thoracic Radiology, the American College of Radiology, and RSNA. J Thorac Imaging.

[17] British Society of Thoracic Imaging. Version 2. Thoracic imaging in COVID-19 Infection. Guidance for the reporting radiologist. https://www.bsti.org.uk/standards-clinical-guidelines/clinical-guidelines/COVID-19-bsti-statement-andguidance/. Epub 2020 Apr 4

[18] Prokop M, van Everdingen W, van Rees Vellinga T, Quarles van Ufford H, Stöger L, Beenen L (2020). CO-RADS: a categorical CT assessment scheme for patients suspected of having COVID-19-definition and evaluation. Radiology.

[19] Pan Y, Guan H, Zhou S, Wang Y, Li Q, Zhu T (2020). Initial CT findings and temporal changes in patients with the novel coronavirus pneumonia (2019-nCoV): a study of 63 patients in Wuhan, China. Eur Radiol.

[20] Song F, Shi N, Shan F, Zhang Z, Shen J, Lu H (2020). Emerging 2019 novel coronavirus (2019-nCoV) pneumonia. Radiology.

[21] Trovato P, Simonetti I, Rinaldo C, Grimaldi D, Verde F, Lomoro P (2021). COVID-19 integrated imaging: our experience and literature review. Pol J Radiol.

[22] Stanzione A, Ponsiglione A, Cuocolo R, Rumolo M, Santarsiere M, Scotto R (2021). Chest CT in COVID-19 patients: structured vs conventional reporting. Eur J Radiol.

[23] de Jaegere TM, Krdzalic J, Fasen BA, Kwee RM (2020). Radiological Society of North America chest CT classification system for reporting COVID-19 pneumonia: interobservers variability and correlation with RT-PCR. Radiol Cardiothorac Imaging.

[24] Özer H, Kılınçer A, Uysal E, Yormaz B, Cebeci H, Durmaz M (2021). Diagnostic performance of Radiological Society of North America structured reporting language for chest computed tomography findings in patients with COVID-19. Jpn J Radiol.

[25] Abdel-Tawab M, Basha MAA, Mohamed IAI, Ibrahim HM, Zaitoun MMA, Elsayed SB (2021). Comparison of the CO-RADS and the RSNA chest CT classification system concerning sensitivity and reliability for the diagnosis of COVID-19 pneumonia. Insights Imaging.

[26] Inui S, Kurokawa R, Nakai Y, Watanabe Y, Kurokawa M, Sakurai K (2020). Comparison of chest CT grading systems in COVID-19 pneumonia. Radiol Cardiothorac Imaging.

[27] Ciccarese F, Coppola F, Spinelli D (2020). Diagnostic accuracy of North America expert consensus statement on reporting CT findings in patients with suspected COVID-19 infection: an Italian Single Center experience. Radiol Cardiothorac Imaging.

[28] Xu PP, Tian RH, Luo S, Zu ZY, Fan B, Wang XM (2020). Risk factors for adverse outcomes with COVID-19 in China: a multicenter, retrospective, observational study. Theranostics.

