# Supplementary material for: RSNA and BSTI grading systems of COVID-19 pneumonia: comparison of the diagnostic performance and interobserver agreement
Source: BMC Med Imaging. 2021 Oct 4;21:143. doi: 10.1186/s12880-021-00668-3 (PMC8487757; doi:10.1186/s12880-021-00668-3)
Supplement: Supplementary file 1 — Additional file 1. Structured thorax CT report recommended by the BSTI in COVID-19 pneumonia. [file 12880_2021_668_MOESM1_ESM.docx]

Table E1. Structured thorax CT report recommended by the British Chest Imaging Society in COVID-19 pneumonia (17).

| Pattern | Appearance |
| --- | --- |
| Classical COVID-19 (100% compatible) | Lower lobe predominant, peripheral predominant, multiple, bilateral* foci of ground glass opacities (GGOs)  ±  Crazy-paving Peripheral consolidation** Air bronchogram Reverse halo/ perilobular pattern** |
| Possible COVID-19 (71–99% compatible) | Lower lobe predominant mix of bronchocentric and peripheral consolidation Reverse halo /perilobullary pattern** GGO scarce |
| Indeterminate (<70% compatible) | Does not fit into definite, probable or Non-Covid  Manifests above patterns, but the clinical context is wrong, or suggests an alternative diagnosis (e.g. an interstitial lung disease in a connective tissue disease setting) |
| COVID-19 exclusion  (<70% compatible with  another diagnosis) | Lobar pneumonia Cavitating infections Tree-in bud/ centrilobular nodularity Lymphadenopathy, effusions Established pulmonary fibrosis |

*>1 lesion, but could still be unilateral; usually but not universally bilateral

**i.e.organising pneumonia patterns
