# Supplementary material for: RSNA and BSTI grading systems of COVID-19 pneumonia: comparison of the diagnostic performance and interobserver agreement
Source: BMC Med Imaging. 2021 Oct 4;21:143. doi: 10.1186/s12880-021-00668-3 (PMC8487757; doi:10.1186/s12880-021-00668-3)
Supplement: Supplementary file 2 — Additional file 2. Expert consensus statement on reporting of RSNA-recommended chest CT findings related to COVID-19. [file 12880_2021_668_MOESM2_ESM.docx]

Table E2. Expert consensus statement on reporting chest CT findings related to COVID-19 (endorsed by RSNA, ACR, and STR) (16).

| **COVID-19 pneumonia imaging classification** | **Rationale** | **CT Findings** | **Suggested Reporting Language** |
| --- | --- | --- | --- |
| **Typical appearance** | Commonly reported imaging features of greater specificity for COVID-19 pneumonia. | Peripheral, bilateral, GGO with or without  consolidation or visible intralobular lines  (“crazypaving”)  Multifocal GGO of rounded morphology with  or without consolidation or visible intralobular lines (“crazy-paving”)  Reverse halo sign or other findings of organizing  pneumonia (seen later in the disease) | “Commonly reported imaging features of  (COVID-19) pneumonia are present.  Other processes such as influenza pneumonia and organizing pneumonia, as can  be seen with drug toxicity and connective  tissue disease, can cause a similar imaging  pattern.” [Cov19Typ]† |
| **Indeterminate appearance** | Nonspecific imaging features of  COVID-19  pneumonia | **Absence of typical features AND Presence of:**  Multifocal, diffuse, perihilar, or unilateral GGO  with or without consolidation lacking a  specific distribution and are nonrounded or  nonperipheral.  Few very small GGO with a nonrounded and  nonperipheral distribution | “Imaging features can be seen with (COVID-19) pneumonia, though are nonspecific and can occur with a variety of  infectious and noninfectious processes.”  [Cov19Ind]† |
| **Atypical appearance** | Uncommonly or  not reported  features of  COVID-19  pneumonia | **Absence of typical or indeterminate features**  **AND Presence of:**  Isolated lobar or segmental consolidation without GGO  Discrete small nodules (centrilobular, “tree-inbud”)  Lung cavitation  Smooth interlobular septal thickening with  pleural effusion | “Imaging features are atypical or uncommonly reported for (COVID-19) pneumonia. Alternative diagnoses should be  considered.” [Cov19Aty]† |
| **Negative for pneumonia** | No features of  pneumonia | No CT features to suggest pneumonia. | “No CT findings present to indicate pneumonia. (Note: CT may be negative in the  early stages of COVID-19.) [Cov19Neg]† |

Note.—Suggested reporting language includes coding of CT findings for data mining. Associated CT findings for each category are based upon available literature at the time of writing in March 2020, noting the retrospective nature of many reports, including biases related to patient selection in cohort studies, examination timing, and other potential confounders. GGO = ground-glass opacity.

Notes:

1. Inclusion in a report of items noted in parenthesis in the Suggested Reporting Language column may depend upon clinical suspicion, local prevalence, patient status as a PUI, and local procedures regarding reporting.

2. CT is not a substitute for RT-PCR, consider testing according to local recommendations and procedures for and availability of RT-PCR.

GGO = ground glass opacity.

† Suggested coding for future data mining.
